# Impact of cytotoxic T lymphocytes immunotherapy on prognosis of colorectal cancer patients

**DOI:** 10.3389/fonc.2023.1122669

**Published:** 2023-01-16

**Authors:** Yankun Zhu, Mingyao Meng, Zongliu Hou, Wenju Wang, Lin Li, Aoran Guan, Ruotian Wang, Weiwei Tang, Fang Yang, Yiyi Zhao, Hui Gao, Hui Xie, Ruhong Li, Jing Tan

**Affiliations:** ^1^ Department of General Surgery, Yan’an Hospital Affiliated to Kunming Medical University, Kunming, China; ^2^ Key Laboratory of Tumor Immunological Prevention and Treatment in Yunnan Province, Yan’an Hospital Affiliated to Kunming Medical University, Kunming, China; ^3^ Department of Pathology, Yan’an Hospital Affiliated to Kunming Medical University, Kunming, China

**Keywords:** colorectal neoplasm, adoptive immunotherapy, T-lymphocyte, histocompatibility antigens class Ⅰ, lymphatic metastasis

## Abstract

**Background:**

Expansion and activation of cytotoxic T lymphocytes (CTLs) *in vitro* represents a promising immunotherapeutic strategy, and CTLs can be primed by dendritic cells (DCs) loaded with tumor-associated antigens (TAAs) transformed by recombinant adeno-associated virus (rAAV). This study aimed to explore the impact of rAAV-DC-induced CTLs on prognosis of CRC and to explore factors associated with prognosis.

**Methods:**

This prospective observational study included patients operated for CRC at Yan’an Hospital Affiliated to Kunming Medical University between 2016 and 2019. The primary outcome was progression-free survival (PFS), secondary outcomes were overall survival (OS) and adverse events. Totally 49 cases were included, with 29 and 20 administered rAAV-DC-induced CTL and chemotherapy, respectively.

**Results:**

After 37-69 months of follow-up (median, 54 months), OS (P=0.0596) and PFS (P=0.0788) were comparable between two groups. Mild fever occurred in 2 (6.9%) patients administered CTL infusion. All the chemotherapy group experienced mild-to-moderate adverse effects, including vasculitis (n=20, 100%), vomiting (n=5, 25%), nausea (n=17, 85%) and fatigue (n=17, 85%).

**Conclusions:**

Lymphatic metastasis (hazard ratio [HR]=4.498, 95% confidence interval [CI]: 1.290-15.676; P=0.018) and lower HLA-I expression (HR=0.294, 95%CI: 0.089-0.965; P=0.044) were associated with poor OS in the CTL group. CTLs induced by rAAV-DCs might achieve comparable effectiveness in CRC patients compare to chemotherapy, cases with high tumor-associated HLA-I expression and no lymphatic metastasis were more likely to benefit from CTLs.

## Introduction

Colorectal cancer (CRC) is a commonly diagnosed malignancy with high mortality worldwide (1). Conventional therapeutic options include surgery, chemotherapy, and radiotherapy (2, 3). In CRC, postoperative adjuvant chemotherapy has been shown to be effective in improving survival and reducing disease recurrence (4). Recommended regimens include fluorouracil and capecitabine, with or without oxaliplatin (5). Despite the obvious benefits of chemotherapy, the prognosis of patients with late-stage or metastatic CRC remains poor. In addition, patients administered chemotherapy experience a variety of adverse effects, including neutropenia, diarrhea, vomiting, and neurotoxicity (5, 6). Therefore, effective treatment strategies with lower toxicity are urgently required.

With the current understanding of the pathophysiology and immunology of cancer, immunotherapy, with the main features of specificity and low toxicity, has become the most promising treatment strategy for patients with CRC (7, 8). To date, immunotherapeutic strategies used in CRC include immune checkpoint blockade, specific antibody therapy, cellular immunotherapy, cancer vaccine therapy, and other combination strategies such as antiangiogenic agents combined with chemotherapy (7, 9). Cellular immunotherapy refers primarily to adoptive cell therapy, which often involves collecting T cells from a patient’s tumor, lymph nodes, or peripheral blood, expanding and activating them *in vitro*, and transferring them to the patient (10). Several clinical trials of CRC have used chimeric antigen receptor T (CAR-T) cell therapy (11), tumor-infiltrating lymphocyte (TIL) therapy (12), and natural killer (NK) cell therapy (13), with variable responses.

In adoptive cell immunotherapy, it is crucial to activate or modify T lymphocytes to specifically kill tumor cells. To this end, antigen-presenting cells (APCs) can be loaded with tumor-associated antigens (TAAs) as an effective approach to prime T lymphocytes. Dendritic cells (DCs), which are considered professional APCs, have been shown to be capable of generating antitumor cytotoxic T lymphocytes (CTLs) (14). There are generally two strategies to load DC cells with TAAs. One is to use tumor lysate containing both identified and unidentified antigens to directly stimulate DC cells (15–19), and the other is to use genetic engineering methods to introduce DNA or mRNA of defined TAAs into DC cells (20, 21).Studies have confirmed that the latter is more effective (21, 22). Recombinant adeno-associated virus (rAAV) is considered to be an ideal vector for genetic engineering because of its non-integration into host genome and no residue (23). rAAV has been used to transform DCs and activate CTLs with tumor associated antigen in several studies, and its safety and effectiveness have been confirmed (23, 24). CTLs induced by rAAV-DC have been used to treat CRC in a phase I clinical trial, and the safety of this approach has been demonstrated (25).

However, the impact of CTLs induced by rAAV-DC in the treatment of CRC remains unclear. Furthermore, no study has identified factors associated with prognosis of CTLs therapy in CRC. Therefore, this study aimed to explore the impact of rAAV-DC-induced CTLs on prognosis of CRC and to explore factors associated with prognosis.

## Patients and methods

### Study design and patients

This prospective observational study included resectable CRC patients in General Surgery Department, Yan’an Hospital Affiliated to Kunming Medical University between February 2016 and 30 September 2019. Inclusion criteria were: 1) age between 18 and 80 years; 2) resectable colorectal tumor confirmed by postoperative histopathology; 3) Karnofsky performance score (KPS) ≥70 before treatment with adequate hematologic, hepatic, and renal functions (i.e., hemoglobin ≥8 g/dL, platelet count ≥100,000/μL, white blood cell count >3000/μL, serum blood urea nitrogen <25 mg/dL, serum creatinine <1.8 mg/dL, and serum ALT, AST, and TBIL levels ≤1.5-times of the respective upper limits of normal [ULNs]). Exclusion criteria were: 1) autoimmune, uncontrolled cardiovascular or lung diseases; 2) pregnancy or lactation (women); 3) treatment with immunosuppressants.

All patients provided written informed consent, and the study was performed in accordance with the Declaration of Helsinki. The ethics committee of Yan’an Hospital affiliated with Kunming Medical University approved the study protocol (approval no. 2015-049-01).

The patients were divided into CTL group and chemotherapy group according to their treatment strategy. In the CTL group, tumor-associated antigens (TAAs) and human leukocyte antigen (HLA) class I protein were required to be positive (weakly positive, positive, or strongly positive) in specimen by an immunohistochemical assay (26).

### Procedures

Patients in CTL group received treatment by infusion with CTLs generated by transfection with DCs harboring rAAV containing TAAs (25).

### Preparation of dendritic cells

Peripheral blood samples (60–70 mL) were collected and subjected to heparin-anticoagulation in each patient receiving immunotherapy. These samples were added to an equal volume of normal saline. Peripheral blood mononuclear cells (PBMCs) separated by Ficoll gradient centrifugation were cultured in 10-cm plates (4.5×10^7^ cells/plate) in AIM-V medium (Invitrogen Co. Carlsbad, CA) at 37°C in a 6% CO_2_ incubator for 3–4 h. Non-adherent cells (lymphocytes) were obtained by gentle shaking and transferred to a centrifuge tube to generate T cells. Several types of rAAVs carrying specific TAA-cDNA (1.0×10^8^/mL, Immuclin Biomed, Inc. Shenzhen, China) were mixed according to immunohistochemical data. Washed adherent cells were added to mixed rAAVs in AIM-V medium containing GM-CSF (800 IU/mL, Amoytop Biotech Co. Ltd. Xiamen, China) and incubated at 37°C in a 6% CO_2_ incubator for more than 8 h. The medium containing the rAAVs was then removed. AIM-V medium containing GM-CSF (800 IU/mL, Amoytop Biotech Co. Ltd.) and IL-4 (1000 IU/mL, R&D Systems, Inc. Minneapolis, MN) was used to induce monocyte differentiation into DCs at 37°C in a 5% CO_2_ incubator. The cytokine-containing medium was refreshed on days 3 and 5, and TNF-α (20 ng/mL, R&D Systems) was added on day 5. Finally, mature DCs were harvested on day 6.

### Preparation of T lymphocytes

Non-adherent PBMCs (lymphocytes) were transferred to T75 culture flasks and cultured in AIM-V medium at 37°C in a 6% CO2 incubator. IL-2 (20 IU/mL, R&D Systems) was added to activate T lymphocytes. The medium was refreshed every other day, and activated T lymphocytes were harvested on day 6.

### Preparation of cytotoxic T lymphocytes

On day 6, activated T lymphocytes and mature DCs were mixed at a 20:1 ratio and co-cultured in 10-cm culture plates in AIM-V medium supplemented with IL-2 (20 IU/mL) and IL-7 (20 ng/mL, R&D Systems) at 37°C in a 6% CO2 incubator. The medium containing IL-2 and IL-7 was refreshed every other day. Finally, cells were harvested on day 15 and washed three times by centrifugation (1200 rpm/min, 12 min) with normal saline. The suspension volume was adjusted to 200 mL. After counting, the CTL suspension was filtered through a cell sieve (100 mesh) once and transferred to a sterile infusion bag or bottle for infusion.

### Transfusion procedure

After evaluation and testing of the specimen, peripheral blood was collected within 4 weeks after surgery to generate CTLs as described above. The harvested CTLs were then infused for 30 minutes intravenously. Subsequent generation and infusion of CTLs was performed every 15 days for a total of 5 times. After each administration, patients were observed for at least 6 h to identify potential adverse effects.

A modified FOLFOX6 regimen was administered to patients who selected chemotherapy. Dosage and schedule were as follows: oxaliplatin at 100 mg/m^2^, ivgtt (2 h) on day 1; 5-flurouracil at 500 mg/m^2^, ivgtt on days 1-5; calcium folinate at 200 mg/m^2^, ivgtt on days 1-5. The first chemotherapy was performed 3–4 weeks after surgery and was repeated every 4 weeks, for 6 times in total.

Demographic (age and sex) and clinical (tumor subtype, lymphatic metastasis and stage at diagnosis) features were also recorded. Patients were staged according to the 8th AJCC CRC pTNM staging system (27).

### Outcomes

The primary outcome was progression-free survival (PFS), defined as the time interval between the date of surgery and the date of progression or death from the disease. Secondary outcomes included overall survival (OS), adverse events, including fever, hypoleukocytemia, liver dysfunction, renal dysfunction, nausea and vomiting. OS was defined as the time interval between the date of surgery and the date of death from any cause. Disability and adverse events leading to prolonged hospitalization or affecting the ability to work were defined as serious adverse events.

### Follow up

The patients were continuously followed up. Chest and abdomen computed tomography (CT) scans were performed every 3 months. Peripheral blood cell counts, liver function, and renal function were tested after each transfusion. In patients administered CTL transfusion, the immunophenotype of peripheral blood cells was also assessed by flow cytometry after each infusion. Any symptoms considered to be related to treatment were recorded. Follow-up ended in September 2021.

### Statistical analysis

SPSS 22.0 (IBM Corp., Armonk, NY, USA) for Windows and GraphPad prism 8 (GraphPad Software Inc., San Diego, California, USA) were used for statistical analysis. Normally and skewed distributed continuous data were expressed as mean ± SD and median (interquartile range), respectively, and compared by the t-test and Mann Whitney U test, respectively. Categorical data were expressed as number (percentage), and compared by the χ^2^ test.

PFS and OS were estimated by the Kaplan-Meier method and compared by the log rank test. In CTL transfusion group, factors associated with prognosis were defined by Cox proportional hazard regression analysis. HLA-I and CEA levels were dichotomized into high (2+ to 3+) and low (+), and TNM stage was dichotomized into early (I/II) and late (III/IV). The status of lymphatic metastasis was marked as positive or negative. Two-sided P<0.05 was considered statistically significant.

## Results

A total of 49 CRC patients were enrolled in this study, including 29 and 20 in the CTL and chemotherapy groups, respectively. There was no case of drop out or lost to follow up. There were no significant differences in demographic and baseline clinical characteristics between the two groups (**Table 1**).

**Table 1:** Baseline characteristics.

Characteristics	All (*n*=49)	Immunotherapy (*n*=29)	Chemotherapy (*n*=20)	P
Age: *n* (%)				0.491
<65	29 (59.2)	16 (55.2)	13 (65)	
≥65	20 (40.8)	13 (44.8)	7 (35)	
Sex: *n* (%)				0.737
Male	28 (57.1)	16 (55.2)	12 (60)	
Female	21 (42.9)	13 (44.8)	8 (40)	
Tumor subtype: *n* (%)				0.238
Colon cancer	27 (55.1)	18 (62.1)	9 (45)	
Rectal cancer	22 (44.9)	11 (37.9)	11 (55)	
Lymphatic metastasis: *n* (%)				0.923
N0	29 (59.2)	17 (58.6)	12 (60)	
N1/N2	20 (40.8)	12 (41.4)	8 (40)	
Stage at diagnosis: *n* (%)				0.923
I/II	29 (59.2)	17 (58.6)	12 (60)	
III/IV	20 (40.8)	12 (41.4)	8 (40)	
TAA expression: *n* (%)				
CEA		27 (93.1)		
Survivin		26 (89.7)		
MAGEA3		28 (96.6)		
CK19		28 (96.6)		
Her-2		5 (17.2)		
Muc-1		8 (27.6)		
HLA-I expression: *n* (%)		29 (100)		

Patients were staged according to the 8^th^ AJCC CRC pTNM staging system.

**Figure 1:**
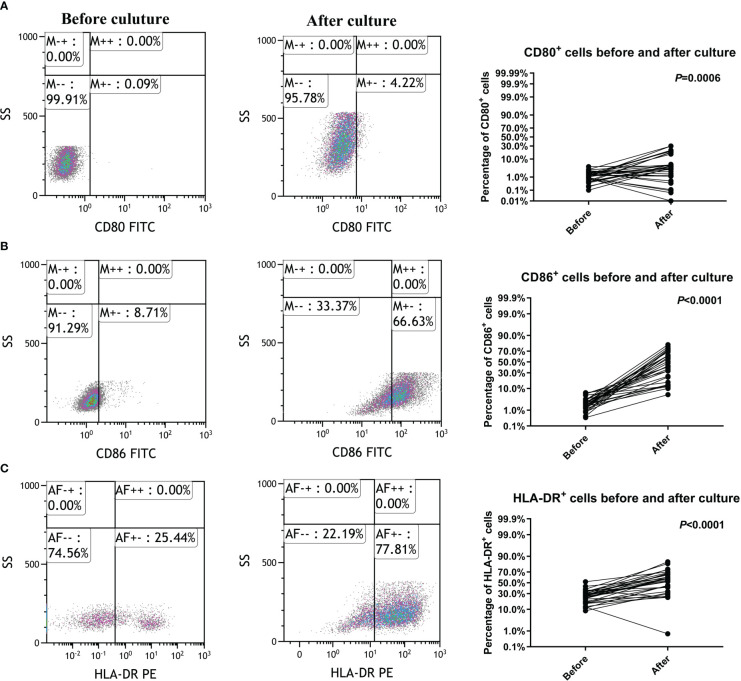
Cytotoxic T lymphocyte activation by culture and induction. **(A)** CD80 was upregulated in Cytotoxic T lymphocytes (CTLs). **(B)** CD86 was upregulated in CTLs. **(C)** HLA-DR was upregulated in CTLs.

After culture, the total average amounts of transfused cells were 3.04 ± 0.25 × 10^8^ cells. The average percentages of CD3+, CD3+CD4+ and CD3+CD8+ lymphocytes were 76.83 ± 2.29%, 37.90 ± 3.84% and 29.72 ± 2.84%, respectively. CD80, CD86 and HLA-DR were upregulated in CTLs after induced by rAAV-DC (**Figure 1**), indicating that the CTLs were indeed activated.

No serious adverse events occurred in either group. Fever occurred in 2 patients administered infusion therapy with CTLs, with temperatures of 38.2°C and 38.7°C, respectively. Both fevers resolved within 6 hours after indomethacin administration. However, all 20 patients administered chemotherapy experienced adverse events, including vasculitis (20 cases, 100%), vomiting (5 cases, 25%), nausea (17 cases, 85%) and fatigue (17 cases, 85%).

**Figure 2:**
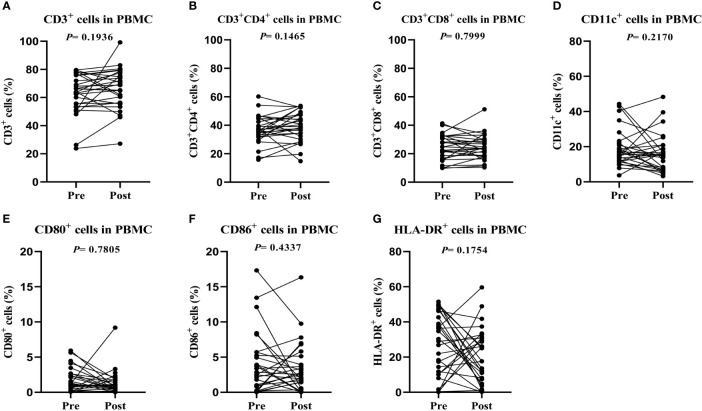
Peripheral blood samples have similar phenotypes before and after cytotoxic T lymphocyte infusion. **(A)** T lymphocyte proportions in peripheral blood. **(B)** Cytotoxic T lymphocyte proportions in peripheral blood. **(C)** Dendritic cell proportions in peripheral blood. **(D–G)** Activated T lymphocyte proportions in peripheral blood. Immunophenotypes were determined by flow cytometry.

The patients were followed up for 37 to 69 months, with a median follow-up duration of 54 months. One sample from the CTL group was excluded due to hemolysis. Twenty-eight pairs of PBMC samples were collected from CTL group patients and analyzed by flow cytometry before and after CTL transfusion. There were no significant differences in the percentages of CD3+, CD3+CD8+, CD11c+, CD80+ and CD86+ cells (**Figure 2**).

**Figure 3:**
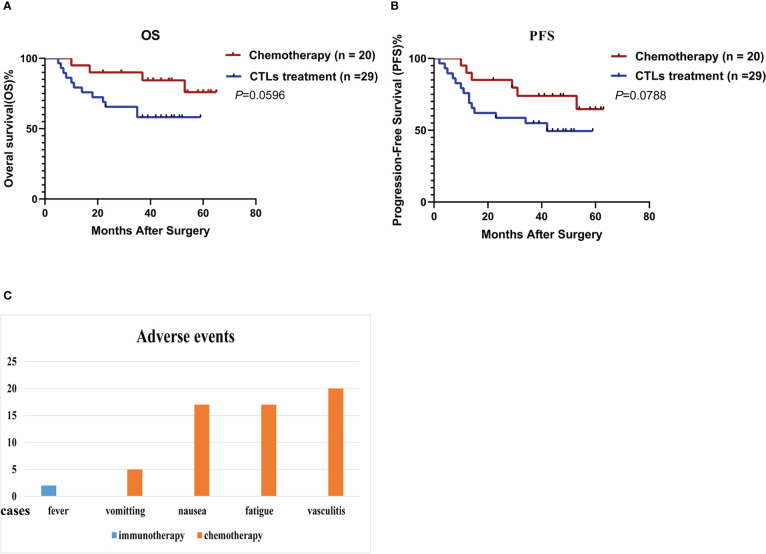
Cytotoxic T lymphocytes induced by rAAVs are effective and well-tolerated. **(A)** Overall survival (OS) and **(B)** progression-free survival (PFS) estimated by the Kaplan-Meier method. **(C)** Adverse events in both group.

Median PFS in the CTL transfusion group was 42 months; the median PFS of the chemotherapy group has not been reached. The 1-, 3- and 5-year PFS rates were 75.9%, 55.0% and 49.5%, respectively, in the CTL group, versus 90%, 74.0% and 64.7%, respectively, in the chemotherapy group. OS rates in the CTL infusion and chemotherapy groups were 58.6% (17/29) and 80.0% (16/20), respectively. The median OS of both groups has not been reached. The 1-, 3-, and 5-year OS rates were 65.5%, 58.2% and 58.2%, respectively, in the CTL group, versus 95%, 90% and 75.9%, respectively, in the chemotherapy group. There were no significant differences in OS (P=0.0596) and PFS (P=0.0788) between two groups (**Figure 3**).

**Figure 4:**
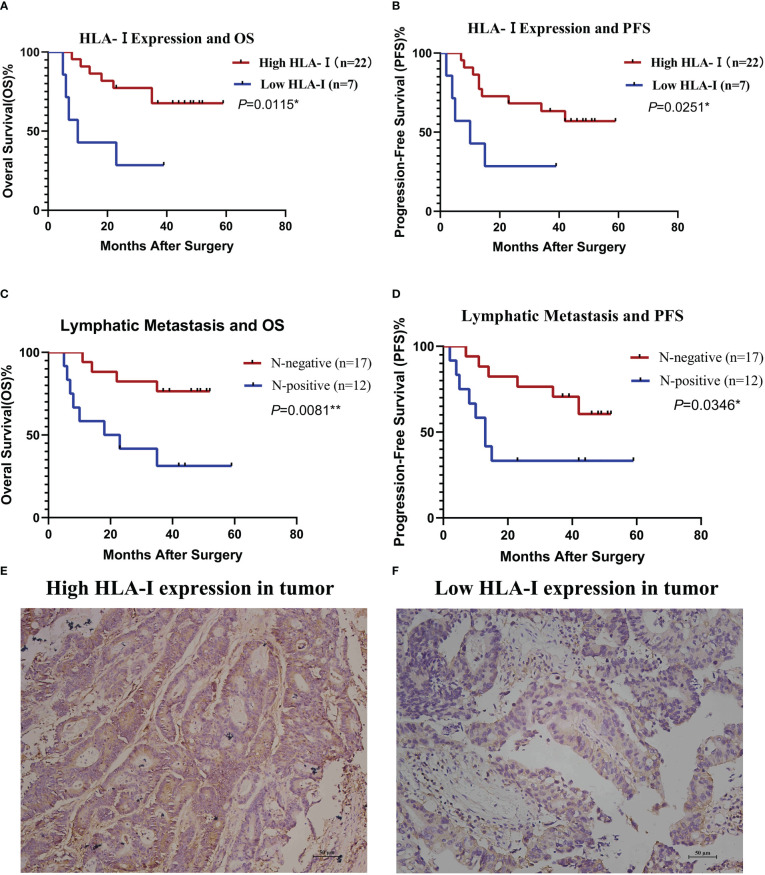
HLA-I expression and lymphatic metastasis are associated with the clinical outcome of cytotoxic T lymphocyte (CTL) infusion therapy. Stratified analysis of overall survival (OS, **A**) and progression-free survival (PFS, **B**) based on HLA-I expression. Stratified analysis of OS **(C)** and PFS **(D)** based on lymphatic metastasis status. OS and PFS were estimated by the Kaplan-Meier method. *P<0.05, **P<0.01. **(E)** Immunohistochemistry of a colon cancer tissue immunostained with anti- HLA-I antibodies showing high HLA-I expression. **(F)** immunohistochemistry of a colon cancer tissue immunostained with anti- HLA-I antibodies showing low HLA-I expression.

In CTL transfusion group, Kaplan-Meier method showed patients with high tumor HLA-I expression had better OS (P=0.0115) and PFS (P=0.0251) compared with those with low tumor HLA-I expression (**Figures 4A, B**), and patients with lymphatic metastasis had worse OS (P=0.0081) and PFS (P=0.0346) compared with those without lymphatic metastasis (**Figures 4C, D**). Cox proportional hazard regression indicated lymphatic metastasis (hazard ratio [HR]=4.498, 95% confidence interval [CI]: 1.290-15.676; P=0.018) and lower HLA-I expression (HR=0.294, 95%CI: 0.089-0.965; P=0.044) were associated with poor OS in the CTL group (**Table 2**) (**Figures 4E, F**).

**Table 2:** Univariate and multivariate analyses of overall survival and progression-free survival in patients administered CTL infusion.

Characteristics	Overall survival				Progression-free survival			
	Univariate analysis		Multivariate analysis		Univariate analysis		Multivariate analysis	
	HR (95%CI)	P	HR (95% CI)	P	HR (95%CI)	P	HR (95%CI)	P
Age (<65 vs. ≥65)	2.087 (0.661-6.586)	0.210			1.013 (0.963-1.065)	0.622		
Gender (Male vs. Female)	0.859 (0.272-2.710)	0.796			1.326 (0.465-3.785)	0.598		
Primary tumor location (Colon vs. Rectum)	0.993 (0.299-3.302)	0.991			0.939 (0.313-2.818)	0.911		
TNM stage (I/II vs. III/IV)	2.611 (0.782-8.719)	0.119			2.140 (0.713-6.424)	0.175		
Lymphatic metastasis (Positive vs. Negative)	4.853 (1.434-16.426)	0.011*	4.498 (1.290-15.676)	0.018*	2.997 (1.027-8.741)	0.044*	2.767 (0.930-8.234)	0.067
HLA-I expression level (+ vs. ++/+++)	0.257 (0.081-0.817)	0.021*	0.294 (0.089-0.965)	0.044*	0.298 (0.097-0.919)	0.035*	0.331 (0.105-1.042)	0.059
CEA expression level (-/+ vs. ++/+++)	1.039 (0.281-3.848)	0.954			1.264 (0.352-4.542)	0.719		

*P < 0.05. HR, hazard ratio; CI, confidence interval.

## Discussion

In this study, rAAV was used to load DCs with defined TAAs according to the immunohistochemical test results of tumor specimens. These rAAV-DCs were further induced to mature cells capable of expressing and presenting TAAs through culture with GM-CSF and IL-4. Then expanded T lymphocytes were activated by rAAV-DCs *in vitro*. After transfusion, T lymphocytes as effector cells could kill tumor cells *in vivo*, while rAAV-DCs could act as tumor vaccine. The results showed there was no significant differences in OS and FPS between CTL transfusion group and chemotherapy group. But the occurrence rates of adverse events were lower in CTL transfusion group. This study also showed that lymphatic metastasis and tumor HLA-I expression were independent prognostic factors in CRC patients administered CTL immunotherapy. The result might provide evidence for application of CTL immunotherapy. Previous studies focused on loading DCs with TAAs to make tumor vaccines, but did not expand and activate T lymphocytes *in vitro* (15, 16, 18–20). Our study focused on the preparation of effector T lymphocytes to maximize tumor killing effect. In addition, we have followed up the patients for more than three years and have a relatively objective evaluation on the therapeutic effect of rAAV-DC-induced CTLs, which is an important supplement to the previous phase I clinical trial focusing on safety research (25).

CRC, a heterogeneous disease caused by many genetic and epigenetic alterations, can be categorized into hypermutated tumors (mutation rates of >12 per 10^6^ base pair) and non-hypermutated tumors based on exome sequencing (28). Since hypermutated tumors may present more neoantigens to the host immune system, they are more sensitive to immunotherapy (29). CRC can also be divided into tumors with mismatch-repair (MMR) deficiency or high levels of microsatellite instability (dMMR-MSI-H) and those with MMR proficiency or low levels of microsatellite instability (pMMR-MSI-L) (28). CRC patients with dMMR-MSI-H do not benefit from fluorouracil-based adjuvant chemotherapy (30). Conversely, such cases are sensitive to immune checkpoint inhibition therapy (31, 32). Due to the limited sample population, the number of cases enrolled are generally small, with the proportion of hypermutated tumor amounting to only approximately 16% of all cases (28). Therefore, this study did not perform sample sequencing or case grouping. Nonetheless, this study showed no significant differences in PFS between the immunotherapy and chemotherapy groups. Further case grouping would have revealed some differences.

CTLs are effector cells of the adaptive immune response. The degree of T lymphocyte infiltration, especially CD8+ T lymphocyte infiltration in tumors, is associated with prognosis in patients with CRC (33). Therefore, many immunotherapeutic strategies focus on improving the functional status of CTLs, which are mostly effective (7). In agreement, the present study found no significant differences in OS and PFS between the CTL and chemotherapy groups. In addition to effectiveness, safety must be considered for clinical application. Immunotherapy can cause a spectrum of adverse events called immune-related adverse events (irAEs) due to the activation of the immune system, which could damage healthy tissues of organs; the incidence of irAEs for immune check point inhibitors is between 23% and 83% (34). In CAR-T cell therapy, a systemic inflammatory response termed the cytokine release syndrome (CRS) is the most common severe irAE; neurological toxicity can also be caused by CAR-T cells (35). Compared with other strategies, there are fewer irAEs following exposure to CTLs induced by rAAV-DCs. DCs and CTLs are both derived from PBMCs collected from patients or their immediate family members; thus, the possibility of immune rejection is very low. rAAV has been approved by the US Food and Drug Administration for clinical use, with proven safety. Activated CTLs kill tumor cells specifically and to a limited extent, with no evidence of a CRS. In this trial, only 2 cases (6.9%) experienced a mild fever. In another previous phase I clinical trial evaluating rAAV-DC-induced CTLs for the treatment of CRC, 27 patients were administered CTL infusion, with cases experiencing fever and fatigue; the overall incidence of irAE was 18.5% (25). The current study and the latter trial demonstrated that rAAV-DC-induced CTL is a safe treatment strategy for CRC patients postoperatively. No significant changes were found in the immunophenotype of PBMCs from the treated patients, which may be due to the dilution of the infused CTLs.

To kill tumor cells, CTLs must distinguish them from normal cells firstly through the binding of T cell receptors (TCRs) on T cells to TAA peptide complexes with tumor cell-specific HLA class I molecules presented on the cell surface. Tumor cells can alter the expression of HLA class I to escape immune surveillance through a variety of mechanisms, e.g., deficient synthesis of β2-microglobulin, loss of genes encoding heavy chains of HLA antigens, mutations that inhibit HLA antigen transcription, and defects in one or more components of antigen processing machinery components (APM) (36). Tumor HLA class I expression is essential for treatment response in patients receiving immunotherapy with CTLs. This study showed that high HLA-I expression was associated with better PFS in CRC patients treated postoperatively with rAAV-DC induced CTLs. Previously, tumor patients with high HLA- I expression were shown to benefit from CTL-based immunotherapy after assessing the responses of tumor cells with different levels of HLA- I expression to CTLs (26). The above data provide evidence confirming this hypothesis in a clinical setting.

Lymph node metastasis was independently associated with worse PFS in CRC patients treated with CTLs in this study. It is well-known that regional lymph nodes are the sites where DCs present tumor-derived antigens to naïve T cells to generate CTLs (37). The theoretical function of lymph nodes is to inhibit metastasis growth, although recent investigation has reported that tumor cell infiltration into lymph nodes induces a certain level of T cell expansion (38). To escape immune surveillance, the tumor can produce a variety of regulatory molecules to downregulate the immune function of lymph nodes (37). In other words, lymphatic metastasis indicates that tumor cells are resistant to CTLs in certain circumstances. This explains why CRC patients with lymphatic metastasis do not benefit from CTL-based immunotherapy.

## Conclusion

This study had several limitations. First, this was a single-center observational study without randomization, which could result in bias. In addition, the sample size was relatively limited, which precluded subgroup analysis. For example, loss of HLA-I is known to induce resistance to immunotherapy, and HLA-I genotype’s diversity may also affect the efficiency of immunotherapy (39). However, due to the limited sample size, we did not perform further HLA typing. Finally, follow-up was relatively short in this study, the OS and PFS was not reached. Therefore, long-term follow-up and large multicenter, randomized controlled studies are needed to verify the effectiveness and safety of CTLs induced by rAAV-DCs. Further analyses based on genotyping are also needed to explore the ideal predictors of response to treatment.

In conclusion, CTLs induced by rAAV-DCs might achieve comparable effectiveness in CRC patients compare to chemotherapy, cases with high tumor-associated HLA-I expression and no lymphatic metastasis were more likely to benefit from CTLs.

## Data availability statement

The original contributions presented in the study are included in the article/supplementary material. Further inquiries can be directed to the corresponding authors.

## Ethics statement

The studies involving human participants were reviewed and approved by Medical Ethics Committee of Kunming Yan’an Hospital (Approval No. 2015-049-01). The patients/participants provided their written informed consent to participate in this study.

## Author contributions

Conceptualization: RL, JT. Methodology: YYZ, MM, WW, FY, HG. Formal analysis and investigation: ZH, WW, LL, AG, RW, HX. Writing - original draft preparation: YKZ, MM. Funding acquisition: RL. Resources: JT. Writing - review and editing and supervision: RL, JT. All authors contributed to the article and approved the submitted version.
